# Intrafamilial Variability in *NRXN1*-Associated Neurodevelopmental Disorders: Clinical and Genetic Insights from a Family Case Study with Literature Review

**DOI:** 10.3390/ijms27146241

**Published:** 2026-07-13

**Authors:** Nikolina Kastratovic, Marina Gazdic Jankovic, Marina Miletic Kovacevic, Sandra Nikolic, Dragica Pavlovic, Dijana Perovic, Vladimir Janjic, Biljana Ljujic

**Affiliations:** 1 Department of Genetics, Faculty of Medical Sciences, University of Kragujevac, 69 Svetozar Markovic Street, 34000 Kragujevac, Serbia; n_kastratovic@outlook.com (N.K.); sandranikolic1972@gmail.com (S.N.); dragica.miloradovic8@gmail.com (D.P.); bljujic74@gmail.com (B.L.); 2 Department of Histology and Embryology, Faculty of Medical Sciences, University of Kragujevac, 69 Svetozar Markovic Street, 34000 Kragujevac, Serbia; marina84kv@gmail.com; 3 Department of Human Genetics, Faculty of Medicine, University of Belgrade, 11000 Belgrade, Serbia; dijanaperovic@gmail.com; 4 Department of Psychiatry, Faculty of Medical Sciences, University of Kragujevac, 69 Svetozar Markovic Street, 34000 Kragujevac, Serbia; vladadok@yahoo.com; 5 Psychiatry Clinic, University Clinical Center Kragujevac, Zmaj Jovina 30, 34000 Kragujevac, Serbia

**Keywords:** neurexin, neurodevelopment, chromosomal microarray, intrafamilial phenotypic variability

## Abstract

The neurexin1 gene (*NRXN1*) encodes a presynaptic adhesion molecule that plays a critical role in synapse formation, maintenance, and function. Copy-number variants (CNVs) affecting the *NRXN1* locus, including submicroscopic deletions, represent rare variant acting as a predisposition for neurodevelopmental disorders, such as Pitt–Hopkins-like syndrome type 2 (MIM #614325) and susceptibility to schizophrenia (MIM #621407). Variations in *NRXN1* gene are associated with marked clinical heterogeneity. We present a familial case involving two male siblings (aged 6 and 5 years) and their 28-year-old mother, all exhibiting variable neurodevelopmental phenotypes. Both children demonstrated disharmonic developmental profiles characterized by impaired communication, speech largely intelligible only to their parents, and behaviors consistent with autism spectrum disorder, including reduced eye contact. Mother represents a carrier with only subtle, nonspecific behavioral traits, further supporting the concept of incomplete penetrance and variable expressivity associated with this genetic alteration. Genetic analysis identified a 317 kb *NRXN1* deletion shared by all affected family members, accompanied by significant intrafamilial phenotypic variability, suggesting the contribution of additional genetic and/or modifying factors. These findings support the concept that *NRXN1* deletions alone do not determine clinical outcome but rather act within a broader genetic and biological context. The marked intrafamilial phenotypic variability and incomplete penetrance observed in this family is compatible with a multiple-hit model, whereby *NRXN1* deletions act as susceptibility factors whose phenotypic consequences are shaped by additional genetic and modifying influences. However, the genetic mechanisms underlying the observed phenotypic variability warrant further investigation.

## 1. Introduction

**Figure 1:**
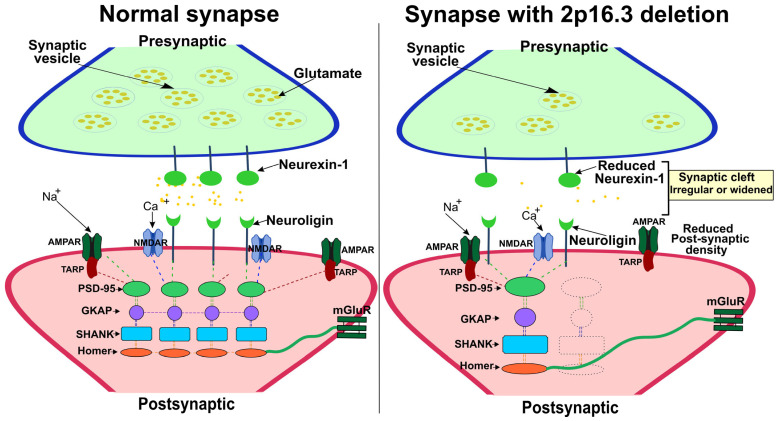
Comparison of a normal glutamatergic synapse and a synapse affected by a 2p16.3 NRXN1 deletion. (**Left**) At a normal glutamatergic synapse, a dense pool of glutamate-filled synaptic vesicles is maintained at the presynaptic active zone. Neurexin-1 bridges the synaptic cleft via interaction with postsynaptic neuroligins, ensuring precise synaptic alignment and efficient neurotransmission. Released glutamate activates postsynaptic AMPAR and NMDAR, mediating Na^+^ and Ca^2+^ influx, respectively. AMPAR function is regulated by its auxiliary subunit TARP. A robust PSD scaffold (PSD-95/GKAP/SHANK/Homer) organizes receptor clustering and signal transduction, while perisynaptic mGluR is anchored to the scaffold via Homer. (**Right**) NRXN1 deletion results in reduced presynaptic Neurexin-1, weakening of the trans-synaptic adhesion complex, decreased vesicle density, and impaired glutamate release. The PSD becomes thin and immature, with partial depletion of scaffold components and reduced AMPAR and NMDAR expression. The synaptic cleft appears irregular or widened. Dashed outlines indicate absent or depleted components. Abbreviations: AMPAR, α-amino-3-hydroxy-5-methyl-4-isoxazolepropionic acid receptor; Ca^2+^, calcium ion; GKAP, guanylate kinase-associated protein; mGluR, metabotropic glutamate receptor; Na^+^, sodium ion; NMDAR, N-methyl-D-aspartate receptor; NRXN1, neurexin-1; PSD, postsynaptic density; PSD-95, postsynaptic density protein 95; SHANK, SH3 and multiple ankyrin repeat domains protein; TARP, transmembrane AMPA receptor regulatory protein.

Neurexins are a family of presynaptic cell-adhesion molecules that play a critical role in synapse formation, maintenance, regulation, and function [1]. The three neurexin genes (*NRXN1*, *NRXN2* and *NRXN3*) are subject to extensive alternative splicing, generating numerous isoforms whose expression varies across different brain regions and developmental stages. Each gene gives rise to two principal isoforms, *NRXN-α* and *NRXN-β*, which differ in the structure of their extracellular domains and in their binding affinities to postsynaptic partners, such as neuroligins. This molecular diversity enables precise regulation and specificity of synaptic connections among neuronal populations [2]. Among these genes, *NRXN1*, located on chromosome 2 at 2p16.3, encodes a protein that functions as a cellular adhesion molecule and receptor within the vertebrate nervous system [3]. *NRXN1* contributes to the organization of neuronal networks connected by synapses, which form the structural basis of neurodevelopment (Table 1) [4]. *NRXN1* interacts with postsynaptic neuroligins to form a molecular bridge, ensuring proper alignment of synaptic structures and facilitating efficient neurotransmission [3]. Loss of *NRXN1* disrupts presynaptic molecular organization, leading to loss of stability and precision, as well as altered coupling between Ca^2+^ channels and vesicle release sites, ultimately resulting in impaired synaptic transmission and network instability (Figure 1). Therefore, due to their critical role in the development, function, and plasticity of neuronal circuits, disruptions in neurexin-encoding genes can result in significant neurodevelopmental disorders [5,6,7].

The NRXN1 locus spans approximately 1.1 Mb and is particularly prone to recurrent copy-number variants (CNVs), which are structural changes in DNA where segments of the gene are either deleted or duplicated [5]. NRXN1 deletions are characterized by variable penetrance and expressivity, resulting in considerable heterogeneity of clinical outcomes, ranging from a broad spectrum of neurodevelopmental disorders such as autism spectrum disorder, intellectual disability, developmental delay [1,8], and attention-deficit/hyperactivity disorder (ADHD), to neuropsychiatric conditions including schizophrenia, depression, anxiety, bipolar disorder, and epilepsy [3,9,10]. Identical or similar deletions may be associated with distinct neurodevelopmental phenotypes, reflecting a complex relationship between genotype and phenotype [11]. Predicting phenotypic outcomes, both within families and among individuals, requires consideration of additional genetic variants and individual differences [12]. Moreover, isoform-specific effects of NRXN1 deletions may contribute to treatment-resistant anxiety, depression, and epilepsy in some individuals, highlighting the need for genotype-targeted interventions [13,14]. In addition, several missense mutations in NRXN1-β (S14L, R8P, T40S and L13F) were associated with autism spectrum disorders. Notably, relatives of patients with these missense mutations did not meet criteria for autism, but exhibited different learning problems and behavioral abnormalities, suggesting variable expressivity of NRXN1-associated phenotypes [15].

Despite the established importance of neurexins in neurodevelopment, the precise mechanisms by which NRXN1 alterations lead to specific behavioral phenotypes remain incompletely understood, limiting the development of effective therapeutic strategies. Thus, understanding the molecular and functional consequences of these alterations may facilitate the development of more precise and personalized therapeutic strategies.

## 2. Results

**Figure 2:**
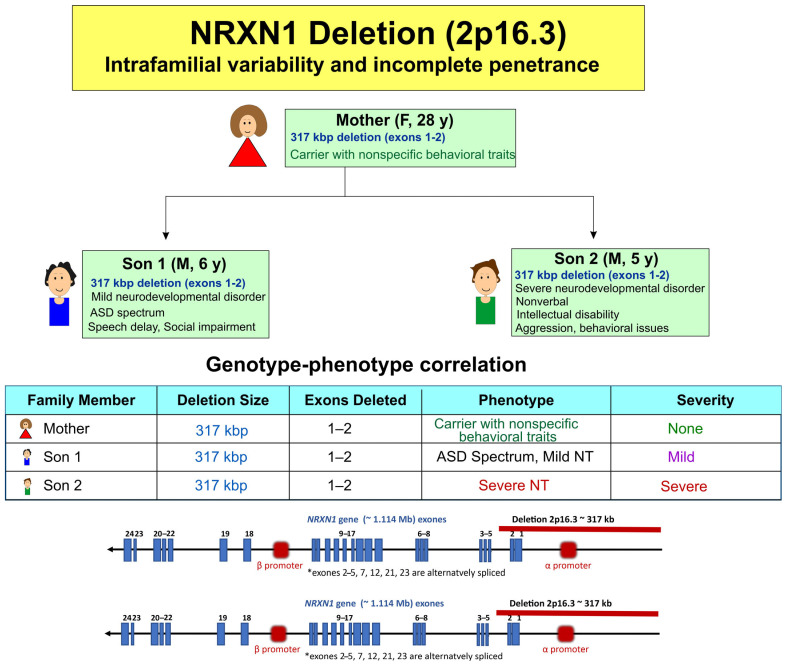
Inheritance and exact position of NRXN1 deletion with phenotypic variability.

The aim of this study is to provide a detailed account of the diagnostic and genetic investigation of a family in which two 6- and 5-year-old boys (Figure 2) were identified with discordant developmental profiles by a neurologist.

### 2.1. Cases Presentation

#### 2.1.1. Patient 1

Patient 1 was the first child born from an uncomplicated pregnancy at 41 weeks of gestation, with regular antenatal follow-up and no detected biochemical or morphological abnormalities. Delivery was vaginal, and the perinatal period was uneventful. Patient 1 was breastfed for 7–8 months, achieved independent sitting at 6 months of age and began walking at 13 months. His first words appeared at 15–16 months. However, between 18 months and 3 years of age, a noticeable regression in speech and behavior was observed. Toilet training was completed at 2.5 years of age. Rehabilitation therapy with speech-language pathologists and special education teachers was started at the age of 2 years. The rehabilitation provided developmental improvement; however, his developmental milestones remain below those expected for his age. He currently attends two weekly sessions with both therapists involved in his rehabilitation.

At the age of 6 years, Patient 1 was referred for genetic evaluation due to disharmonic development, characterized by limited communication skills, speech intelligible only to parents, difficulties with attention maintained primarily through interaction with a phone, mild aggressive tendencies, and stereotyped behaviors. At the time of examination, his body weight was 23.8 kg (89th percentile), height was 120 cm (91st percentile), and occipitofrontal head circumference was 53 cm (83rd percentile).

Neurodevelopmental milestones showed overall preserved fine motor skills, with independence in dressing, bathing, and putting on shoes, as well as good precision in manual tasks. Learning abilities were difficult to fully assess due to behavioral features; however, the clinical presentation was consistent with autism spectrum disorder traits. Speech was functional for basic needs, although intelligibility was limited in broader communicative contexts, with mild improvement in vocabulary expansion reported by speech therapy follow-up.

At the current evaluation, Patient 1 demonstrated brief and inconsistent eye contact, poor cooperation during examination, impaired social interaction and signs of distress, including anxiety, fearfulness, tearfulness, and episodes of irritability and aggression. Behaviorally, Patient 1 presented with stereotyped movements and obsessive behaviors, partially reduced under therapeutic intervention. Audiological evaluation demonstrated normal hearing. No sleep disturbances have been reported. EEG findings were within normal limits, and according to the maternal history, there have been no episodes suggestive of seizures or staring spells. On physical examination, dysmorphic features included an elongated face, prominent ears, and a prominent chin. The neck, thorax, abdomen, and upper extremities were unremarkable, with no visible dysmorphic characteristics. The sacral region was unremarkable. Examination of the lower extremities revealed a bilateral sandal gap deformity and pes planus. External genitalia were consistent with a male phenotype.

#### 2.1.2. Patient 2

Patient 2, the second child from the second pregnancy, was delivered via vaginal delivery at 41 weeks following a regularly monitored pregnancy, which was uneventful with no detected biochemical or morphological abnormalities. This 5-year-old boy was subsequently evaluated due to concern that the phenotype observed in his brother, Patient 1, might have a hereditary component. According to his mother, Patient 2 was breastfed until 2.5 years of age. Early motor milestones were within the expected range, as he was able to sit at 6 months and walk at 13 months. His first words were produced at approximately 14 months. Rehabilitation therapy, including speech and special education therapy, was started at 2 years and 8 months, consisting of one session per week with a special education therapist and four sessions per week with a speech-language pathologist. Patient 2 was independent in basic daily activities, although he presented with multiple functional and behavioral limitations. Fine motor skills and self-care are age-appropriate.

At the time of examination, his body weight was 16.1 kg (42nd percentile), height was 100 cm (22nd percentile), and occipitofrontal head circumference was 51 cm (46th percentile).

The clinical evaluation was partially challenging due to significant behavioral features consistent with autism spectrum disorder. These included reduced and inconsistent eye contact, occasional stereotyped movements, and obsessive behaviors, accompanied by episodes of irritability and mild aggression. Attention span was limited and was often sustained only with the use of a mobile phone as an external focus of engagement.

Language abilities were impaired, with speech that was comprehensible to parents but largely unintelligible to unfamiliar listeners. Despite observed developmental progress, Patient 2’s overall development remains below age expectations, with the ability to form simple sentences. No sleep disturbances have been reported. Neurological investigations, including EEG, were within normal limits. According to maternal history, there have been no episodes suggestive of seizures or staring spells.

At the physical examination, the patient was noted to have a long facial shape, a depressed nasal bridge, and a broad mouth, consistent with previously described findings. The neck, thorax, abdomen and upper extremities showed no visible deformities or dysmorphic characteristics. Examination of the lower extremities revealed bilateral sandal gap. External genitalia were consistent with a male phenotype.

#### 2.1.3. Patient 3

Patient 3 was the mother of patients 1 and 2. She spoke at the age of 3. Apart from delayed speech development, she shows no evidence of intellectual disability or other significant neurological or psychiatric disorders. Clinical evaluation was unremarkable except for self-acknowledged obsessive personality traits. (Table 2).

We analyzed the frontal images of patients 1 and 2 using the Face2Gene application (https://www.face2gene.com), which automatically suggests syndromes as a tentative diagnosis based solely on facial gestalt. The typical features associated with *NRXN1* included craniofacial dysmorphisms, deep-set eyes, broad nasal bridge, bulbous nose tip, flat mid-face, ear defects and wide mouth.

**Figure 3:**
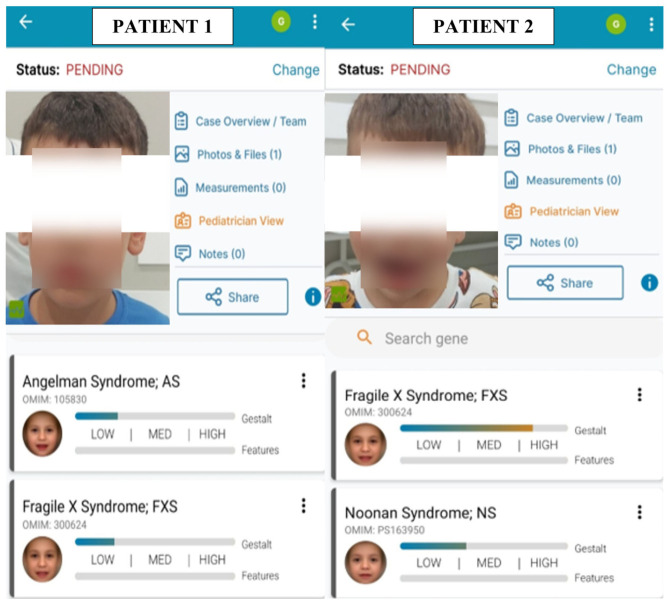
Representative Face2Gene phenotyping results demonstrating automated prediction of the most probable genetic syndromes based on facial morphological features.

When analyzing only the frontal image, Face2Gene application did not suggest the correct diagnosis. However, it identified syndromes that were also of neurodevelopmental origin (Fragile X, Angelman, Noonan) indicating partial overlap with the observed clinical phenotype (Figure 3).

#### 2.1.4. Genetic Analysis and Interpretation

**Figure 4:**
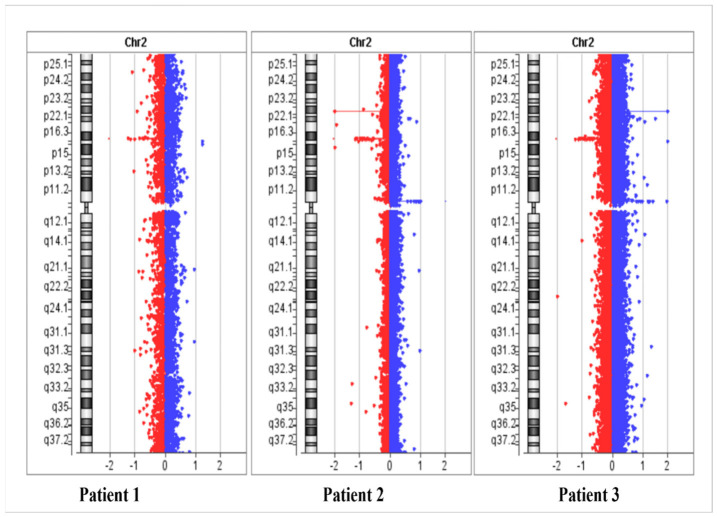
Results of array-CGH of Patient 1, Patient 2 and Patient 3. Zero value indicates an equal fluorescence intensity ratio between the sample and reference. Copy number losses shifted the ratio toward the left side (red), whereas copy number gains shifted it towards the right side (blue). Our results demonstrate a deletion in the short arm of chromosome 2 (2p16.3, 317 kb) in all three patients.

High-resolution molecular karyotyping in both siblings revealed a heterozygous deletion in the 2p16.3 region, approximately 317 kb in size, encompassing the 5′ upstream region and first two (NM_001330078.2) or three exones (NM_001135659.3) specific to the α-isoform of the NRXN1 gene (MIM 600565). The deletion was classified as pathogenic (GRCh37: 2p16.3(51193626_51510961) × 1) according to ACMG/ClinGen criteria [16]. Subsequent parental testing revealed that the deletion was inherited from the mother with a mild, unremarkable phenotype (Patient 3) (Figure 4, Supplementary Figure S1). *NRXN1* is known to be haploinsufficient, with a ClinGen HI score of 3. The partial overlap of the 5′ end of an established HI gene (3′ end of the gene not involved) with the coding sequence involved, along with an increased variant frequency in cases compared to controls, supports the classification of the gene deletion as pathogenic, although there is recognized decreased penetrance and variable expressivity associated with the deletion. (https://search.clinicalgenome.org/kb/gene-dosage/HGNC:8008 accessed on 5 November 2025).

## 3. Discussion

The *NRXN1* gene is essential for proper synaptic development, function and organization, and its derangement has been linked to a wide range of neurodevelopmental disorders, including speech and language delays, cognitive disability and autism-related traits [6]. Although CNVs are relatively rare, when they occur in *NRXN1*, they are considered among the strongest single-gene genetic risk factors for neurodevelopmental disorders. Changes at the molecular level, such as deletions or duplications, can disturb synaptic organization and reduce stability of neuronal connections. Synapses become less precisely aligned, neurotransmission becomes less efficient, and overall neuronal communication is weakened, which can contribute to broader synaptic-level dysfunction in the brain and increase vulnerability to conditions affecting brain development and function [5].

Differences in clinical presentation and genetic heterogeneity suggest that the effects of *NRXN1* deletions depend on the size of deletion and specific genomic regions affected [5,17]. Diagnosed patients typically harbor heterozygous deletions involving only a portion of the coding region, most frequently affecting the promoter and initial exons encoding the *NRXN1α* isoform [10,12,18], which may have variable effects on neurexin expression and function, and consequently on phenotypic outcomes [19,20]. *NRXN1α* deletions are linked to intellectual disability, epilepsy, autism, language and speech delays. In contrast to *NRXN1α*, deletions of *NRXN1β* are less common but are generally associated with more severe phenotypes, including moderate to severe intellectual impairment, language delays, epilepsy, and autistic traits [12]. Recent studies indicate that the effects of *NRXN1* deletions are dependent on cell type and developmental stage, contributing to variability in clinical manifestations. These findings further support the need for individualized therapeutic approaches [10,11]. Heterozygous *NRXN1* deletions are associated with neurodevelopmental disorders including autism spectrum disorders, intellectual disability, ADHD, and neuropsychiatric conditions, but display reduced penetrance and variable expressivity.

Compound heterozygous mutations can lead to more severe phenotypes resembling Pitt–Hopkins syndrome type 2 (MIM #614325). In this family, the deletions correlate with disharmonic development, language delays, and behavioral features, consistent with the reported spectrum of *NRXN1*-associated phenotypes. *NRXN1* compound heterozygous variants have been associated with mild facial dysmorphism, including frequent associations with wide mouth, persistent tongue protrusion, hypersalivation, and strabismus [5,6]. Mono-allelic functional variants of NRXNs have also been identified in healthy controls, suggesting that additional genetic, epigenetic, or environmental factors may influence the penetrance and phenotypic expression of NRXN deficiency (multiple-hit) [5,21]. However, bi-allelic *NRXN1* deletions are rare and can result in a more severe phenotypes with profound cognitive and severe developmental impairment, absent speech, social communication difficulties, abnormal repetitive motor activity, early-onset epilepsy, gastroesophageal reflux, sleep–wake dysregulation, hypotonia and constipation [1,5,22,23].

Different sizes and locations of *NRXN1* deletions, particularly those involving specific exons of the *NRXN1-α* isoform, have been associated with variable neurodevelopmental phenotypes. Zahir et al. (2008) reported a de novo heterozygous 320 kb deletion at chromosome 2p16.3, encompassing the *NRXN1-α* promoter and exons 1–5 while sparing the *NRXN1-β* promoter, in a patient presenting with mild intellectual disability, autistic features, vertebral abnormalities, and dysmorphic facial characteristics, highlighting the importance of proper *NRXN1-α* dosage for normal neurological development [24]. In one large patient cohort study conducted by Schaaf et al. (2012), deletions ranging from 17 to 913 kb and affecting between 2 and 13 exons demonstrated considerable phenotypic heterogeneity [25]. Exonic deletions were consistently associated with developmental delay/intellectual disability, autism spectrum disorder, hypotonia, seizures, speech impairment, attention-deficit/hyperactivity disorder, and varying degrees of dysmorphism [25]. In addition, in another cohort study Dabell et al. (2013) identified 27 patients with *NRXN1* deletions among 30,065 tested individuals [26]. The detected deletions ranged from 40 to 586 kb and demonstrated substantial phenotypic variability, even though the majority affected the *NRXN1-α* isoforms [26]. Nevertheless, several core clinical manifestations were consistently observed among most individuals, including developmental delay, speech impairment, abnormal behavior with autistic features or autism spectrum disorder, and varying degrees of dysmorphic features [26]. These findings further support the association between the size and localization of *NRXN1* deletions, particularly those involving specific *NRXN1-α* exons and the severity as well as the spectrum of neurodevelopmental manifestations. Cooper et al. reviewed 12 studies on *NRXN1* mutations, including 25 cases with exonic and intronic deletions. Most of those deletions affect the first five exons, and only three involve only the first two exons, with or without the upstream region of the alpha promoter, as in ours [20].

In a manner similar to our findings, Harrison et al. (2011) described variable penetrance within the same family carrying *NRXN1* alterations, reporting 2 sisters with compound heterozygous deletions at chromosome 2p16.3 exclusively involving the *NRXN1* gene [22]. Both sisters exhibited a severe neurodevelopmental phenotype, including early-onset epilepsy, profound intellectual disability, hypotonia, and significantly delayed motor development [22]. Nevertheless, even within the same family and despite a shared genetic background, the clinical presentation differed between the sisters, particularly regarding respiratory manifestations, neurological symptoms and developmental milestones. Additional clinical features observed in both patients included stereotypic behavior, disturbed sleep–wake patterns, gastroesophageal reflux associated with poor growth, constipation, precocious puberty and scoliosis [22]. These observations illustrate the remarkable clinical variability and incomplete penetrance associated with *NRXN1* deletions, even among closely related individuals.

Detecting such genomic alterations often requires high-resolution genetic analyses, such as advanced molecular karyotyping or whole-exome sequencing, to uncover subtle or partial deletions. Failure to accurately diagnose these variants can delay interventions, restrict access to targeted therapies and postpone prognostic counseling for affected individuals and their families. Accurate identification of partial *NRXN1* deletions within a family is particularly important, as these variants often display reduced penetrance and variable expressivity, making phenotype prediction challenging [3]. Combining family history, clinical assessment and high-resolution molecular karyotyping allows reliable detection and monitoring of carriers. Precise identification of submicroscopic variants not only clarifies inheritance patterns but also aids in anticipating potential neurodevelopmental outcomes in offspring, serving as a crucial diagnostic and preventive tool in clinical practice while minimizing the risk of misdiagnosis.

The phenotypic variability observed in this family, particularly between the mother and her children, is consistent with the well-documented reduced penetrance and variable expressivity associated with *NRXN1* deletions [11,20]. Although the same genetic alteration was identified, the clinical manifestations differ, supporting previous findings that *NRXN1*-associated phenotypes cannot be predicted solely based on genotype. This variability likely reflects the influence of additional genetic, epigenetic and environmental factors that modulate phenotypic expression [11,20]. At the molecular level, the differences in clinical presentation may be related to variation in the functional impact of the deletions, including the specific exons involved and their effect on *NRXN1* isoform expression. Such differences may lead to variable disruption of synaptic function, which could account for the distinct neurodevelopmental profiles observed among affected family members [17,27].

Both children from our study should receive regular follow-up with a child psychiatrist due to potential neuropsychiatric risks. Developmental assessments and therapy, including speech and special education interventions, should be continued and intensified. Whole-exome sequencing (WES) is recommended to investigate additional *NRXN1*-related variants and syndromic associations. Pitt–Hopkins-like syndrome 1 (MIM #610042) can also be caused by homozygous or compound heterozygous variants and/or intragenic deletions in the *CNTNAP2* (contactin-associated protein-like 2) gene located on chromosome 7q35–q36.1. Affected individuals present with a variable clinical spectrum characterized by neurodevelopmental delay with intellectual impairment, early-onset epilepsy, loss of expressive language abilities and abnormal respiratory manifestations [28]. Absence of WES is a limitation of the present study, since it could preclude a more comprehensive assessment of additional genetic contributors that may influence the observed phenotypical difference among the family members. Neurexin-associated disorders are increasingly considered within a multiple-hit model, in which the primary pathogenic variant may interact with other genetic or environmental modifiers to shape clinical presentation. Therefore, the presence of secondary genetic variants potentially contributing to intrafamilial phenotypic variability observed in family cannot be excluded.

Incomplete penetrance and variable expressivity are well-recognized characteristics of NRXN1-related disorders and may partly explain the marked heterogeneity among carriers of the same variant. The observation of mother as unaffected carrier in this study highlights the phenomenon of incomplete penetrance, indicating that the presence of a CNVs alone may not be sufficient to cause disease manifestation. Instead, the variant may act as a predisposing factor whose clinical effects depend on additional genetic, epigenetic, or environmental modifiers. This concept is further supported by the marked phenotypic variability observed among affected individuals carrying alterations in the same gene. The variability in clinical manifestations observed within our family, ranging from nonspecific behavioral traits to more pronounced neurodevelopmental impairment, likely reflects the complex interplay between the identified variants and additional modifying factors that were beyond the scope of the current study. Consequently, identical or similar genetic variants may result in a broad spectrum of clinical outcomes. Such variability complicates genotype–phenotype correlations and suggests that the clinical expression of neurodevelopmental disorders is likely influenced by a multifactorial interplay of contributing factors beyond the primary pathogenic variant. The co-segregation of the *NRXN1* deletion within the family presented in this study further supports the clinical relevance of this variant and its association with a broad spectrum of neurodevelopmental manifestations, while the observed phenotypic differences among carriers illustrate the variable expressivity previously described for *NRXN*-associated conditions [29,30].

Therefore, the intrafamilial variability observed in this case studies may be consistent with a multiple-hit model, in which *NRXN1* deletions alone may not be sufficient to determine the clinical phenotype but instead act in combination with additional genetic or modifying factors [19,31]. Nevertheless, this hypothesis could not be evaluated in the present study because of the lack of WES, and therefore the potential contribution of additional genetic variants to the observed intrafamilial phenotypic variability remains unknown. This model provides a plausible explanation for the differing severity and presentation of symptoms within the same family and highlights the importance of comprehensive genetic evaluation in such cases. Additionally, this work demonstrates marked intrafamilial variability, as different individuals within the same family carrying the same or similar genetic alterations may present with distinct clinical manifestations.

## 4. Materials and Methods

### 4.1. Study Design

This investigation was designed as a family-based case report, incorporating a retrospective analysis of clinical and molecular genetic data. The study included a family consisting of a 6-year-old boy, a 5-year-old boy and their mother (28 years old). Prior to the diagnostic evaluation, the family had no significant personal, familial, or hereditary history relevant to this study.

#### 4.1.1. Patient Consent and Ethical Approval

Written informed consent for publication of this case report was obtained from the parents. Ethical approval was obtained from the local ethics committee of Faculty of Medical Sciences University of Kragujevac, Serbia (No. 09-2082/16, 6 March 2026).

#### 4.1.2. Molecular Karyotyping

Molecular genetic analysis was performed using high-resolution chromosomal microarray (CMA) analysis with the Agilent SurePrint G3 Human CGH array kit 4x180K + SNP (UCSC hg19, NCBI Build 37, February 2009) (Agilent Technologies, Santa Clara, CA, USA) according to manufacturer’s protocol. Data were analyzed using CytoGenomics software version 5.4 (Agilent Technologies).

### 4.2. Facial Analysis Using the Face2Gene Platform for Syndrome Identification

The Face2Gene application, a DeepGestalt-based framework (FDNA, Boston, MA, USA), is a next-generation phenotyping technology used to identify genetic syndromes from facial features and was applied in this study to detect dysmorphic features associated with *NRXN1* deletion.

## 5. Conclusions

The intrafamilial variability highlights the complex relationship between genotype and phenotype and suggests that the impact of *NRXN1* deletions is influenced by additional genetic and environmental factors, including potential synergistic interactions with other genes. A careful and detailed genetic analysis is a key necessity in the diagnosis of neurodevelopmental syndromes that are not easily recognizable based on clinical presentation alone.

## Figures and Tables

**Table 1:** Comparison of different synaptic features depending on *NRXN1* presence.

FEATURE	NORMAL SYNAPSE	2p16.3 (*NRXN1*) DELETION
Adhesion	Strong (NRXN1-NLGN complex)	Weak or absent
Vesicle pool	High density at active zone	Reduced or disorganized
Synaptic cleft	Uniform width	Irregular or widened
Post-synaptic density	Robust and mature	Thin and immature
Functional outcome	Efficient signal transmission	Impaired connectivity

**Table 2:** Genotype–phenotype correlation.

	Patient 1	Patient 2	Patient 3
Early development and medical history	Abnormalities detected (regression in speech at 18 months)	Abnormalities detected (delayed milestones in speech)	Abnormalities detected (spoke at the age of 3)
Fine motor skills and self-care	Mild	No abnormalities detected	No abnormalities detected
Cognitive and learning profile	Moderate (behavior is consistent with features associated with autism spectrum disorder)	Severe (behavioral features consistent with autism spectrum disorder, including episodes of anger and mild aggression)	No abnormalities detected
Speech and communication	Mild (verbal communication is sufficient for basic needs, though clarity and comprehensibility are reduced in broader social and communicative situations)	Severe (verbal output is understood to a limited extent by the parents; however, speech is largely unintelligible to others)	No abnormalities detected
Behavior and social interaction	Moderate	Severe	Mild
Sleep	No abnormalities detected	No abnormalities detected	No abnormalities detected
Neurological status	Moderate (disharmonic development)	Moderate (disharmonic development)	No abnormalities detected

Face2Gene evaluation.

## Data Availability

The data presented in this study are available in this article and Supplementary Material.

## Supplementary Materials

The following supporting information can be downloaded at: https://www.mdpi.com/article/10.3390/ijms27146241/s1.
