# Supplementary figures and images for: Intrafamilial Variability in NRXN1-Associated Neurodevelopmental Disorders: Clinical and Genetic Insights from a Family Case Study with Literature Review
Source: Int J Mol Sci. 2026 Jul 13;27(14):6241. doi: 10.3390/ijms27146241 (PMC13409882; doi:10.3390/ijms27146241)

A

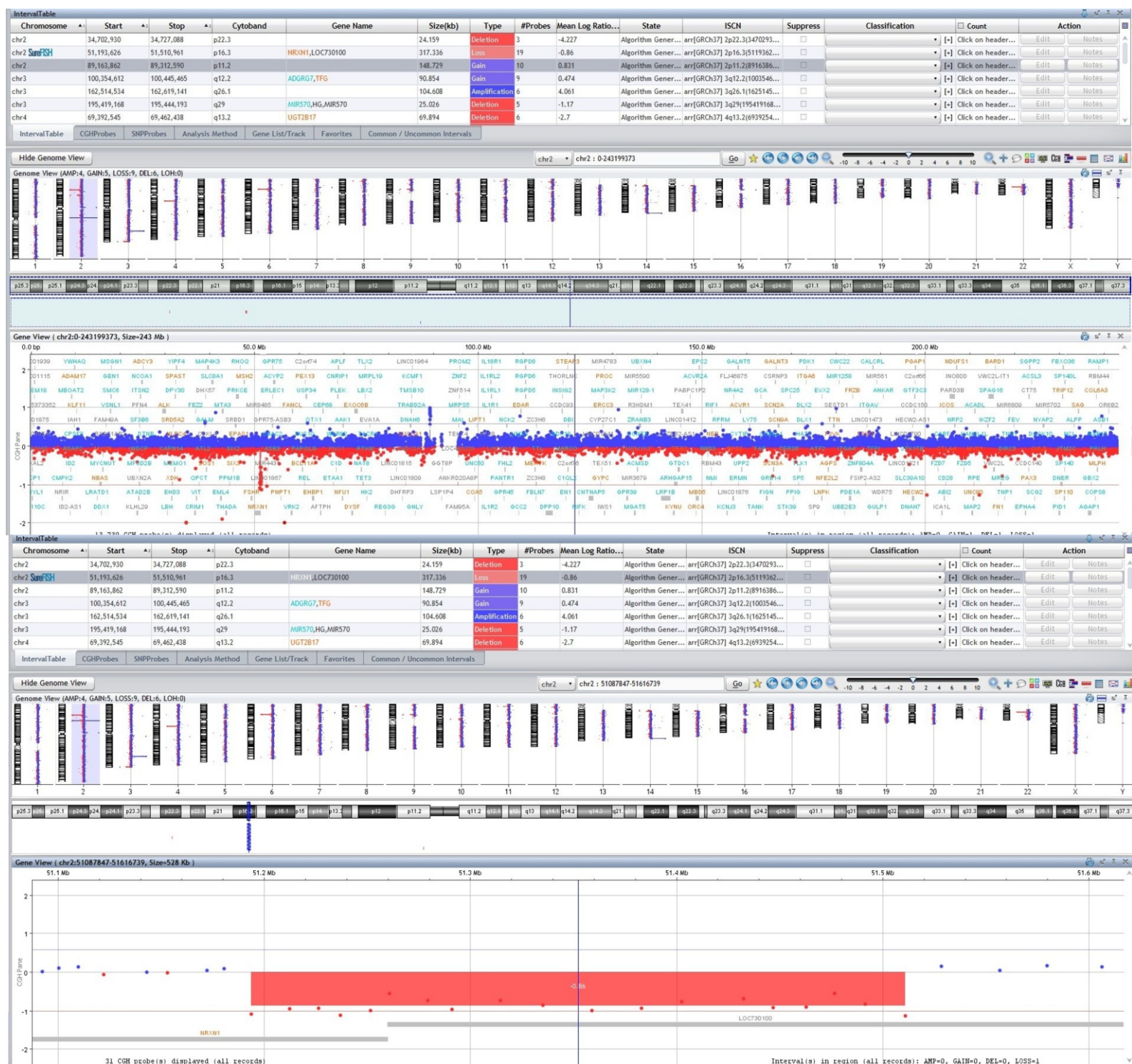

B

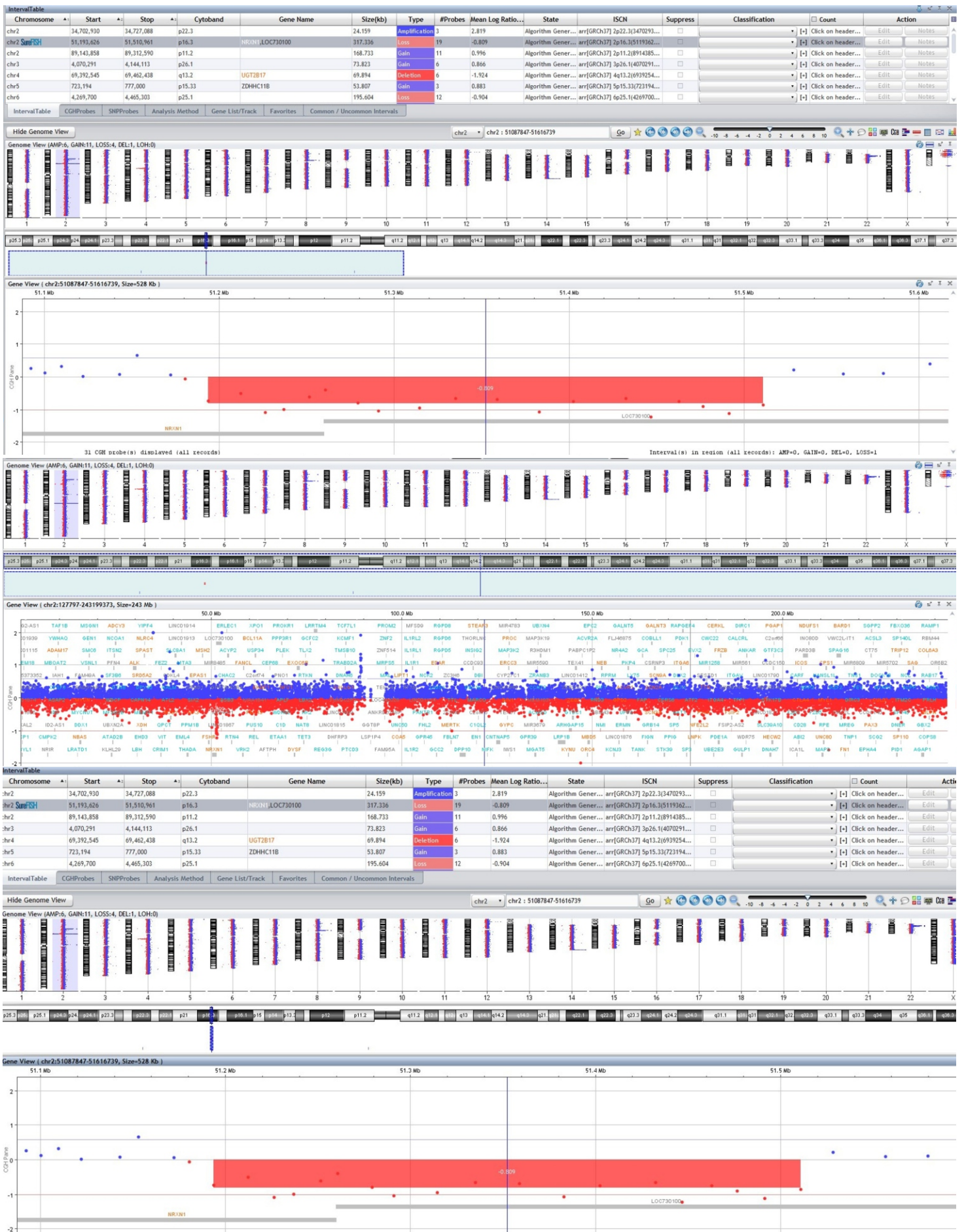

Supplement: Supplementary file 1 [file ijms-27-06241-s001.zip › ijms-4384342-supplementary.pdf]
